# Behavioral problems in migrant children born preterm: Is it about language or is it about the system?

**DOI:** 10.1111/dmcn.16242

**Published:** 2025-01-20

**Authors:** Frank Müller, Christine Happle, Eva Noack

**Affiliations:** ^1^ Department of General Practice University Medical Center Göttingen Göttingen Germany; ^2^ Department of Family Medicine, College of Human Medicine Michigan State University Grand Rapids MI USA; ^3^ Department of Pediatric Pneumology, Allergology, and Neonatology Hannover Medical School Hannover Germany

## Abstract

**This commentary is on the original article by Jaekel et al. on pages** 600–608 of this issue.

Preterm birth affects millions of children globally, and providing equitable care poses particular challenges in increasingly diverse societies. The study by Jaekel et al. convincingly demonstrates a correlation between children's first language linguistic distance to German and behavioral and emotional problems in children born preterm. [Correction added on 18 February 2025 after first online publication: The author's name Jaeckel was changed to Jaekel in the preceding sentence.] This interrelation is especially relevant in Germany where, as the authors point out, about 42% of children have at least one immigrant parent and preterm birth affects approximately 1% to 2% of all infants.^1^

A central question arises. Do these findings primarily reflect language differences and communication problems that emerge from them, or do they indicate broader systemic challenges in early childhood education, childcare systems, and pediatric care? The actual German language proficiency of children or their parents was not assessed in the study. However, the authors assume that greater linguistic distance of the primary learned language results in lower German language proficiency, making it more difficult for both children and parents to navigate society. When children struggle with communication, this may cascade into frustration or feelings of isolation, increasing their risk for behavioral and emotional problems. Similarly, parents may face barriers to accessing and effectively utilizing available support services.

However, the presumed language barriers might also serve as a proxy for a range of intersectional factors and deeper structural inequities faced by immigrant families, particularly those from Turkish or Arabic‐speaking backgrounds.

According to a recent federal report, individuals with Turkish migration backgrounds face a significantly higher poverty risk compared to both native Germans and other migrant groups (https://www.bpb.de/kurz‐knapp/zahlen‐und‐fakten/sozialbericht‐2024/553284/erwerbs‐haushaltseinkommen‐und‐armutsrisikoquote/). While medical care for many children born preterm in Germany is coordinated in specialized pediatric care clinics providing regular follow‐up visits, their access to early childhood education through kindergarten and pre‐K institutions is less stringently supported. In Germany, children from migrant families are less likely to attend preschool institutions.^2^ These institutions are widely available and facilitate the development of children's socio‐emotional, cognitive, physical, and communication skills. It is recognized that early childhood education promotes educational equity as it can compensate for unfavorable developmental conditions such as household poverty, low literacy, or post‐immigration stress through specific socio‐pedagogical support.^3^

However, this requires sufficient investment in social infrastructure. According to OECD figures, Germany spends only 1.1% of its GDP on early childhood education (compared to Norway and Iceland which spend 1.9% and 2.0% respectively). For primary school education, Germany ranks second to last among all OECD countries: 0.7% of GDP (while Israel invests 2.6%).^4^ Both unions and business associations lament the shortage of qualified daycare staff and daycare places, with current estimates suggesting a shortage of up more than 400 000 daycare spots.^5^

Given these structural deficits, it would be misguided to attribute behavioral problems to ‘language barriers’ or ‘cultural differences’ only. Such narratives ignore systemic shortcomings and risk fueling racist and xenophobic sentiments.

Adequate staffing, financial resources, and recognition of early childhood educators' professional contributions are key to providing all children with the support they need and fostering multilingualism as an enrichment rather than a burden. For children born preterm from migrant families, comprehensive follow‐up care should extend beyond medical issues to include encouragement of early preschool education. However, research on current practices and their effectiveness is needed.

Without addressing these systemic shortcomings, ‘language barriers’ will likely continue to serve as a marker for broader social inequities affecting immigrant families, especially with children born preterm.

## Data Availability

Not required.
